# Combined clinical and genomic signatures for the prognosis of early stage non-small cell lung cancer based on gene copy number alterations

**DOI:** 10.1186/s12864-015-1935-0

**Published:** 2015-10-06

**Authors:** Ander Aramburu, Isabel Zudaire, María J. Pajares, Jackeline Agorreta, Alberto Orta, María D. Lozano, Alfonso Gúrpide, Javier Gómez-Román, Jose A. Martinez-Climent, Jacek Jassem, Marcin Skrzypski, Milind Suraokar, Carmen Behrens, Ignacio I. Wistuba, Ruben Pio, Angel Rubio, Luis M. Montuenga

**Affiliations:** Group of Bioinformatics, CEIT and TECNUN, University of Navarra, San Sebastian, Spain; Laboratory of Biomarkers, Program in Solid Tumors and Biomarkers, Center for Applied Medical Research, University of Navarra, Pio XII, 55, 31008 Pamplona, Spain; Program in Hemato-Oncology, Center for Applied Medical Research, University of Navarra, Pamplona, Spain; Department of Pathology, Clinica Universidad de Navarra, Pamplona, Spain; Department of Oncology, Clinica Universidad de Navarra, Pamplona, Spain; Department of Pathology, Marques de Valdecilla University Hospital, School of Medicine, University of Cantabria, Santander, Spain; Department of Oncology and Radiotherapy, Medical University of Gdańsk, Gdańsk, Poland; Department of Translational Molecular Pathology, The University of Texas M.D. Anderson Cancer Center, Houston, TX USA; Department of Thoracic/Head and Neck Medical Oncology, The University of Texas M.D. Anderson Cancer Center, Houston, TX USA; Department of Histology and Pathology, School of Medicine, University of Navarra, Pamplona, Spain; Department of Biochemistry and Genetics, School of Sciences, University of Navarra, Pamplona, Spain; Navarra’s Health Research Institute (IDISNA), Pamplona, Spain

**Keywords:** Early stage lung cancer, Prognosis, Copy number profiling, Gene filtering, Semi-supervised learning

## Abstract

**Background:**

The development of a more refined prognostic methodology for early non-small cell lung cancer (NSCLC) is an unmet clinical need. An accurate prognostic tool might help to select patients at early stages for adjuvant therapies.

**Results:**

A new integrated bioinformatics searching strategy, that combines gene copy number alterations and expression, together with clinical parameters was applied to derive two prognostic genomic signatures. The proposed methodology combines data from patients with and without clinical data with a priori information on the ability of a gene to be a prognostic marker. Two initial candidate sets of 513 and 150 genes for lung adenocarcinoma (ADC) and squamous cell carcinoma (SCC), respectively, were generated by identifying genes which have both: a) significant correlation between copy number and gene expression, and b) significant prognostic value at the gene expression level in external databases. From these candidates, two panels of 7 (ADC) and 5 (SCC) genes were further identified via semi-supervised learning. These panels, together with clinical data (stage, age and sex), were used to construct the ADC and SCC hazard scores combining clinical and genomic data. The signatures were validated in two independent datasets (*n* = 73 for ADC, *n* = 97 for SCC), confirming that the prognostic value of both clinical-genomic models is robust, statistically significant (*P* = 0.008 for ADC and *P* = 0.019 for SCC) and outperforms both the clinical models (*P* = 0.060 for ADC and *P* = 0.121 for SCC) and the genomic models applied separately (*P* = 0.350 for ADC and *P* = 0.269 for SCC).

**Conclusion:**

The present work provides a methodology to generate a robust signature using copy number data that can be potentially used to any cancer. Using it, we found new prognostic scores based on tumor DNA that, jointly with clinical information, are able to predict overall survival (OS) in patients with early-stage ADC and SCC.

**Electronic supplementary material:**

The online version of this article (doi:10.1186/s12864-015-1935-0) contains supplementary material, which is available to authorized users.

## Background

Pulmonary resection is the standard treatment for early stage non-small cell lung cancer (NSCLC). The potential benefits of adjuvant chemotherapy (ACT) and/or radiotherapy after surgery have been explored in a large number of clinical trials [1]. After the publication of two meta-analyses that showed a significant 5-year survival improvement of 4 % in patients treated with ACT [2], guidelines from European and American medical societies recommend cisplatinum based ACT in stage II-IIIA patients. Controversial results were obtained in relation to stage IB patients, which show a slight but statistically non-significant improvement in overall survival (OS) after ACT [3]. Adjuvant treatment is not recommended in stage IA patients because clinical trials showed no benefits, and even a decrease in OS after this treatment. Despite the progress in therapy in NSCLC, five-year OS rates are around 65 % for stage I and 40 % for stage II [4]. This is partly due to the fact that the current staging system is not precise enough to stratify the real risk of relapse in early patients. Hence, the discovery and validation of new molecular biomarkers that could classify these early patients in subgroups, to identify those with worse prognosis who could benefit from ACT, is a clear unmet need.

A large number of studies have been developed to define a prognostic genomic signature in early stage lung cancer, most of them based on mRNA expression microarrays [5–7]. However, although clinical parameters are validated predictors for OS, most prognostic profiles do not provide a decision making algorithm combining both the molecular markers and the clinicopathologic features (sex, age, stage, etc.) of each patient.

NSCLC can be divided into three main subclasses: adenocarcinoma (ADC), squamous cell carcinoma (SCC) and large cell carcinoma (LCC); the most common subtypes being ADC and SCC. The need of a robust and reproducible genetic profile is especially apparent for SCC, as most of the genetic prognostic profiles described so far for lung cancer patients are restricted to ADC histology [8]. Indeed, the two commercially available prognostic tests for lung cancer (both RNA-based) are intended for ADC patients and are not valid for SCC [5, 7]. Despite the efforts to characterize prognosis of SCC patients [9–11], the published information has not been translated into a validated clinically useful tool, partly due to the existence of several biological subtypes within the squamous lung tumors [12, 13].

Most of the published prognostic signatures are gene expression (RNA)-based profiles. Considering the superior stability of DNA compared to RNA, a prognostic profile based on the analysis of tumor DNA rather than RNA would most likely achieve a more robust and reproducible clinical applicability due to the higher stability of the DNA. Even though some publications have described copy number aberrations (CNA) as predictors of early stage lung cancer survival, most of them only focus on individual gene or chromosomic region alterations and do not provide a proposal for a signature based on CNA in the context of stage I-II lung cancer [14, 15].

The goal of this study was the generation of two new CNA-based prognostic clinical-genomic signatures for the prognosis of stage I-II separately for ADC and SCC. Data from a total of 632 patients were used. We first analyzed CNA profiles of tumor samples from an initial cohort of 155 (99 ADC and 56 SCC) stage I-II patients from three different datasets. Other series of patients were used to provide additional information to the analysis and for independent validation. First, we identified a subset of candidate genes which fulfilled a double condition: a) positive correlation between copy number and gene expression; and b) correlative association of the expression of the gene with prognostic value according to two publicly available databases. Next, using the CNA for each of the selected genes and the clinical data (if available), we developed the clinical-genomic signature which estimated an individual patient prognosis. Finally, both (ADC and SCC) integrative clinical-genomic signatures were validated using independent series of early stage, non-treated ADC and SCC patients from The Cancer Genome Atlas (TCGA) consortium.

In summary, we describe here a new methodology to derive CNA-based prognostic clinical-genomic signatures and propose two signatures which may be useful in predicting prognosis of stage I-II ADC and SCC. In our analysis we also show the strength of combining both genetic and clinical data in prognostic studies.

## Methods

### Patients

In the training series, we included clinical-genomic data of 632 patients from 5 different datasets: three previously published, GSE28582 [16], GSE25016 [17] and GSE34140 [18], and two unpublished, CIMA-CUN-HUMV and The University of Texas MD Anderson (MDA) Cancer Center. Patients included in these novel datasets gave the required informed consent. All patients underwent surgical resection of NSCLC. Among the 632 specimens included in the datasets, 338 were ADC (99 labeled and 239 unlabeled data) and 294 were SCC (56 labeled and 238 unlabeled data). We considered a sample to be labeled if survival data were available. All labeled patients had early-stage tumors (stage IA, IB, IIA or IIB) and did not receive preoperative or postoperative chemotherapy. We used the unlabeled data (from datasets GSE25016 and GSE34140) as an additional source of information to derive the clinical-genomic models. The median follow up times for ADC and SCC training sets were 61 and 73 months, respectively (Data gathering in Additional file 1). The project has been approved by the Ethical Committee of the University of Navarra on April 22nd 2010, approval ref number 068/2010 and by the MD Anderson Cancer Center Institutional Review Board 1, on December 7th 2013, Protocol number PA13-0230”.

The clinical-genomic signatures for ADC and SCC were validated using two independent datasets obtained from The Cancer Genome Atlas (TCGA; https://tcga-data.nci.nih.gov/tcga/). These datasets corresponded to patients with completely resected stage I or II NSCLC who had not received any type of adjuvant therapy (n = 73 for ADC, and n = 97 for SCC). The median follow up times for these ADC and SCC validation series were 20 and 23 months, respectively (Data gathering in Additional file 1).

### Data processing pipeline

**Fig. 1:**
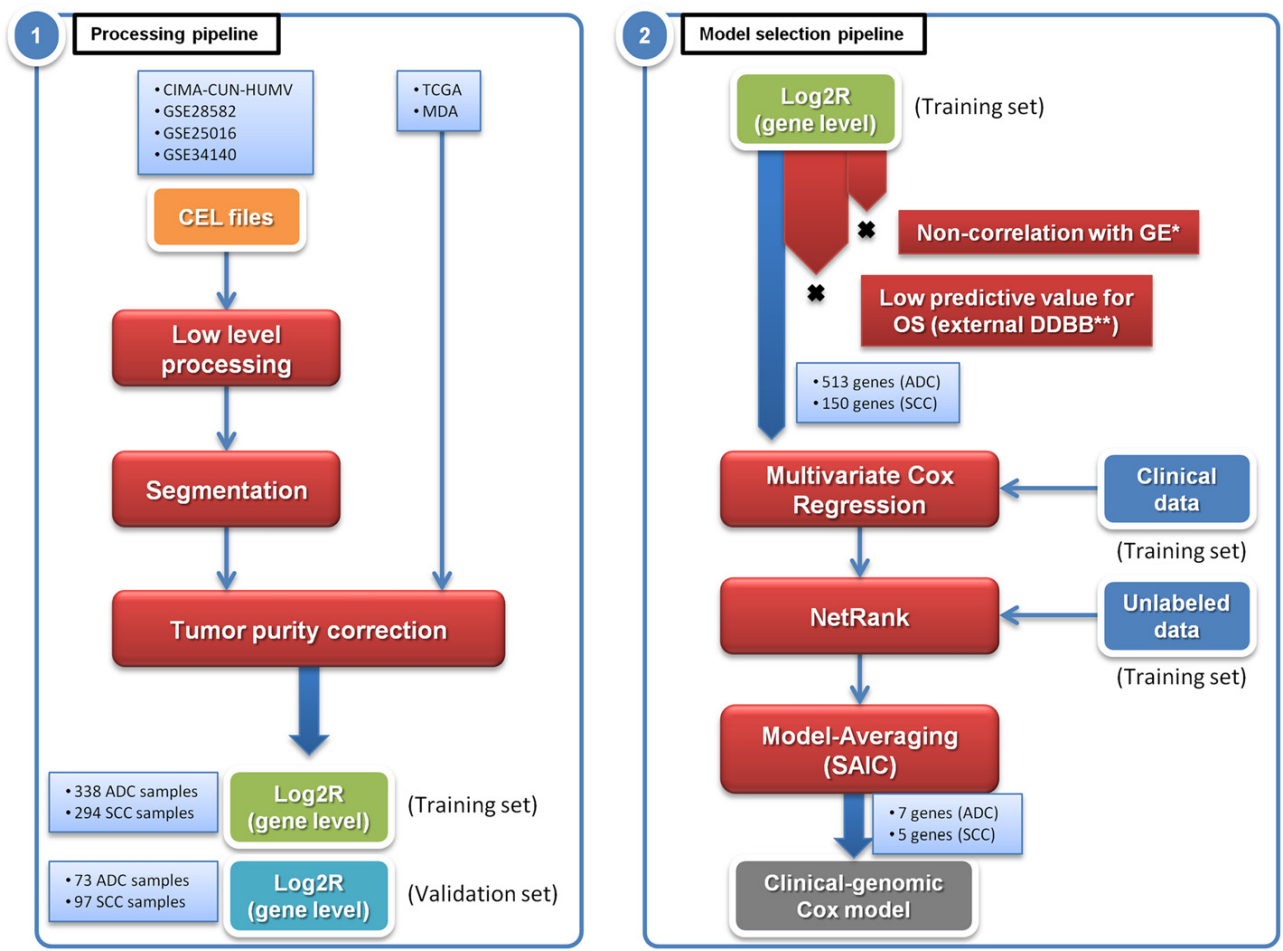
Panel 1, Main processing pipeline steps. Panel 2, Model selection pipeline followed to obtain the final clinical-genomic signatures. *Gene Expression (GE). **Databases (DDBB)

Tumor DNA from CIMA-CUN-HUMV DNA samples was hybridized to Affymetrix 500 K SNP microarrays. Intensities of scanned images were quantified, normalized and summarized using ACNE [19]. Then, total copy number values were estimated using NSA [20]. Since copy number profiling can be affected by stromal contamination, copy number values for each sample were re-scaled according to their tumor purity using GPHMM [21] (Core algorithm in Additional file 1). This correction also reduces variation among samples both within the same dataset, and across datasets. Next, values were segmented using CBS [22] to get regions of constant number of copies. These steps were performed for all datasets, except for TCGA and MDA datasets. For these ones, data were already processed by the providers and the tumor purity values were available, allowing us to perform the correction. Finally, segmented copy number values were assigned to each gene. If copy number changes were found within a gene, a weighted median of the copy number values of its internal segments was assigned (Core algorithm in Additional file 1). For some of the genes, we validated the microarray data using this pipeline by FISH. We found a high coherence between both techniques (data not shown). In Fig. 1, panel 1 illustrates the data processing steps described above. These data have been deposited at the GEO database (http://www.ncbi.nlm.nih.gov/geo/) in a superseries with access numbers GSE72195 (it embraces GSE72192 and GSE72194).

### Model selection pipeline

Due to the biological differences between ADC and SCC, data were analyzed separately. Prior to any analysis, two filters were applied to generate a list of candidate genes. Firstly, a “correlation filter” was used to remove those genes whose copy numbers do not correlate with their expression. Datasets described above, and data available from two additional sets of patients [23, 24] were used for this correlation study. The threshold used to call for a positive correlation was a local FDR adjusted q-value smaller than 0.2. Secondly, a “prognostic filter” was used to remove genes not related to OS based on two external databases: GeneSigDB [25] and Prognoscan [26]. The list downloaded from GeneSigDB contained manually curated genes associated with lung cancer OS (Core algorithm in Additional file 1). To generate the list from Prognoscan, we performed a meta-analysis for each gene across all the available studies included in this database (Core algorithm in Additional file 1). The selected genes for the prognostic filter were those that appeared to be significant in any of the two databases. Our list of candidate genes was thus prepared looking for those genes which shared both positive correlation between copy number and gene expression, and presence in any of the lists of genes with prognostic value from GeneSigDB or Prognoscan. GO pathway analyses of the candidate genes were done for ADC and SCC separately using GeneCoDis software analysis (http://genecodis.cnb.csic.es).

Next, we used a Cox proportional-hazards regression analysis to relate survival with clinical data and CNA for each candidate gene independently using only the training set. The statistical significance of the coefficient associated with the analyzed gene determined the gain in classification power of the survival model compared with the clinical model alone. Only clinical data that have proved to have prognostic value (age, sex and stage) were included. Age was considered to be a continuous variable, sex a dichotomous categorical variable (female was taken as reference), and stage a categorical variable with ordered levels (stages). For the latter, the stages were modeled as incremental risk and stage IA was taken as reference. The absolute hazard ratio for a given stage in our model was obtained by summing up the incremental hazard ratios from earlier stages. In the generation of the survival models, the Cox coefficients associated with age, sex and the incremental risks of the stages were forced to be non-negative. These non-negativity constraints are coherent with previous clinical studies [4].

To select the most relevant genes for OS prediction, additional information retrieved from the unlabeled datasets was incorporated into the model using the NetRank algorithm [27]. The NetRank algorithm mimics the PageRank algorithm that Google uses to rank its results. Each gene has a relevance given by its p-value. Using a network generated with correlations (using unlabeled samples), the initial ranking of the p-values was changed to accommodate the additional information of the network. With the aid of the Akaike Information Criterion (AIC), the algorithm selected an ensemble model that combines the best candidate models (Core algorithm in Additional file 1).

Finally, we generated two mathematical models that predict OS for ADC or SCC. In both models, clinical (age, sex and stage) and gene copy number data were included. Then, patients’ risk scores were calculated and patients were classified as having a high-risk signature or a low-risk signature with the median of the risk scores as threshold value. In Fig. 1, panel 2 illustrates the survival model selection pipeline described above.

### Prognostic evaluation

The risk model was validated on the TCGA dataset (that was not used in the training phase). Using the predicted risk score, the prediction performance was analyzed with a univariate Cox proportional hazards regression taking the corresponding predicted risk scores as explanatory variable. A one-tailed p-value for a hazard ratio less than 0.05 was considered to be significant. Alternatively, all patients in the TCGA dataset were dichotomized into two groups: low-risk and high-risk; and a log-rank test was also performed. The latter is a non-parametric test that evaluates the null-hypothesis that both groups have similar survival. In addition, to carry out a statistical comparison of the prognostic power of the clinical-genomic signatures with the prognostic power of mere clinical signatures, a Harrell’s test was performed [28]. This test compares prediction models and provides information on whether the alternative model is significantly better than the reference model. In our case, we performed two different Harrell’s tests for both ADC and SCC. In the first test, we compared the clinical-genomic test with a reference model that included only clinical variables. In the second, we compared the genetic test with the clinical-genomic one.

## Results

Using lung ADC and SCC data from three labeled and two unlabeled datasets as training sets, we sought to find a consistent gene signature that combined with clinical prognostic factors (age, sex and stage) would model OS risk. A validation dataset for each NSCLC subtype was used to confirm the prognostic value of the clinical-genomic signatures and the improved predictive power of these models compared with the clinical ones.

### Derivation of the clinical-genomic signatures

After the filtering processes (using the correlation and prognostic filters described in Methods), we selected 513 and 150 candidate genes for ADC and SCC, respectively (Additional file 2: Table S6 and Additional file 3: Table S7). In the 513 selected gene set for ADC, the GO biological processes highly significantly enriched were related either to cell proliferation or metabolism. Among the top 15 enriched pathways 13 were related to these two categories, including “RNA metabolic process” (GO:0016070; P = 6.40e-22), “M/G1 transition of mitotic cell cycle” (GO:0000216; *P* = 1.25e-20), “mRNA metabolic process” (GO:0016071; *P* = 1.21e-19), “regulation of cellular amino acid metabolic process” (GO:0006521; P = 5.75e-18), “G1/S transition of mitotic cell cycle” (GO 0000082; *P* = 1,69E-16); “cell cycle checkpoint” (GO: 0000075; *P* = 4,95E-16), etc. On the other hand, intriguingly, in the case of SCC, the selected candidate gene set presented a much smaller number of enriched gene sets and their relationship with cancer pathways is not as apparent. In fact, only two pathways were significantly enriched, which suggest the much higher intrinsic biological heterogeneity in the SCC cases analyzed in the previously published cohorts. The most prominent enriched GO biological pathways in SCC are: “leukemia inhibitory factor signaling pathway” (GO:0048861; *P* = 0.046) and “morphogenesis of a polarized epithelium” (GO:0001738; *P* = 0.046). The complete tables with the enriched gene sets for both types of cancer, ADC and SCC, are included in Additional file 4: Tables S8 and Additional file 5: Table S9, respectively.

Then, a gene ranking was performed using the multivariate Cox regression p-values. The regressors for each gene were the clinical variables and the number of gene copies. Then, this gene ranking was modified based on the NetRank methodology. Finally, several candidate gene signatures obtained according to the Akaike Information Criterion were averaged according to the Akaike weights. This methodology rendered two clinical-genomic signatures (see Table 1) containing 7 and 5 prognostic genes for ADC and SCC, respectively (Detailed calculations and intermediate results are shown in Additional file 1). The genes included in the ADC signature were: *YES1* and *TYMS* (both located at 18p11.32), *HMGN1* (21q22.2), *PSMA4* (15q25.1), *MYO1E* (15q22.2), *POFUT2* (21q22.3) and *SLC25A20* (3p21.31) In the SCC signature the genes selected through our algorithm were: *GPD1L* (located at 3p22.3), *TRA2B* (3q27.2), *CTNND1* (11q12.1), *DICER1* (14q32.13) and *ZNF292* (6p14.3).

**Table 1:** Genes that constitute the 7-gene and 5-gene signature for ADC and SCC

Signature	Gene name	Cytoband	DNA copy number^a^	
			Poor prognosis^b^	Good prognosis^b^
ADC	*YES1*	18p11.32	0.11	−0.18
	*TYMS*	18p11.32	0.11	−0.19
	*HMGN1*	21q22.2	−0.12	0.11
	*PSMA4*	15q25.1	0.01	−0.23
	*MYO1E*	15q22.2	−0.04	−0.19
	*POFUT2*	21q22.3	−0.13	0.10
	*SLC25A20*	3p21.31	−0.08	−0.27
SCC	*GPD1L*	3p22.3	−0.41	−0.24
	*TRA2B*	3q27.2	0.66	0.72
	*CTNND1*	11q12.1	−0.21	0.07
	*DICER1*	14q32.13	0.13	−0.23
	*ZNF292*	6p14.3	0.04	−0.14

^a^Mean gene copy number data (in log2ratio) are shown for the training set

^b^Patients with a risk score greater (smaller) than the median are considered patients with poor (good) prognosis

In relation to the clinical covariates, our methodology imposed their directions based on *a priori* knowledge [4]. In particular, the risk coefficients for age, sex and the incremental risk for each of the stages were forced to be non-negative (i.e., the overall risk was imposed to increase with age and stage and to be higher in men than in women). In our analysis, the coefficients for sex and the incremental risk of stage IIA relative to IB were null in both clinical-genomic signatures (when the restriction of the direction was not included, the risk for these clinical variables had a small negative value -data not shown-). As a result, both clinical-genomic signatures included the same predictive clinical factors: age, stage IB vs IA and stage IIB vs IB (Survival model inference in Additional file 1).

All genes present in the clinical-genomic ADC and SCC signatures, except *SLC25A20*, were significantly associated with survival (*P* < 0.05). In particular, *YES1* and *TYMS* showed the highest predictive power (*P* < 0.001). The predictive power of SCC genes was slightly lower than that of ADC genes. Furthermore, the predictive power of the stage covariates in the SCC model was not statistically significant. These could be due to the reduced sample size of the SCC training set, with only 56 labeled samples, compared with the 99 ADC labeled samples.

### Prognostic evaluation of the clinical-genomic signatures

**Fig. 2:**
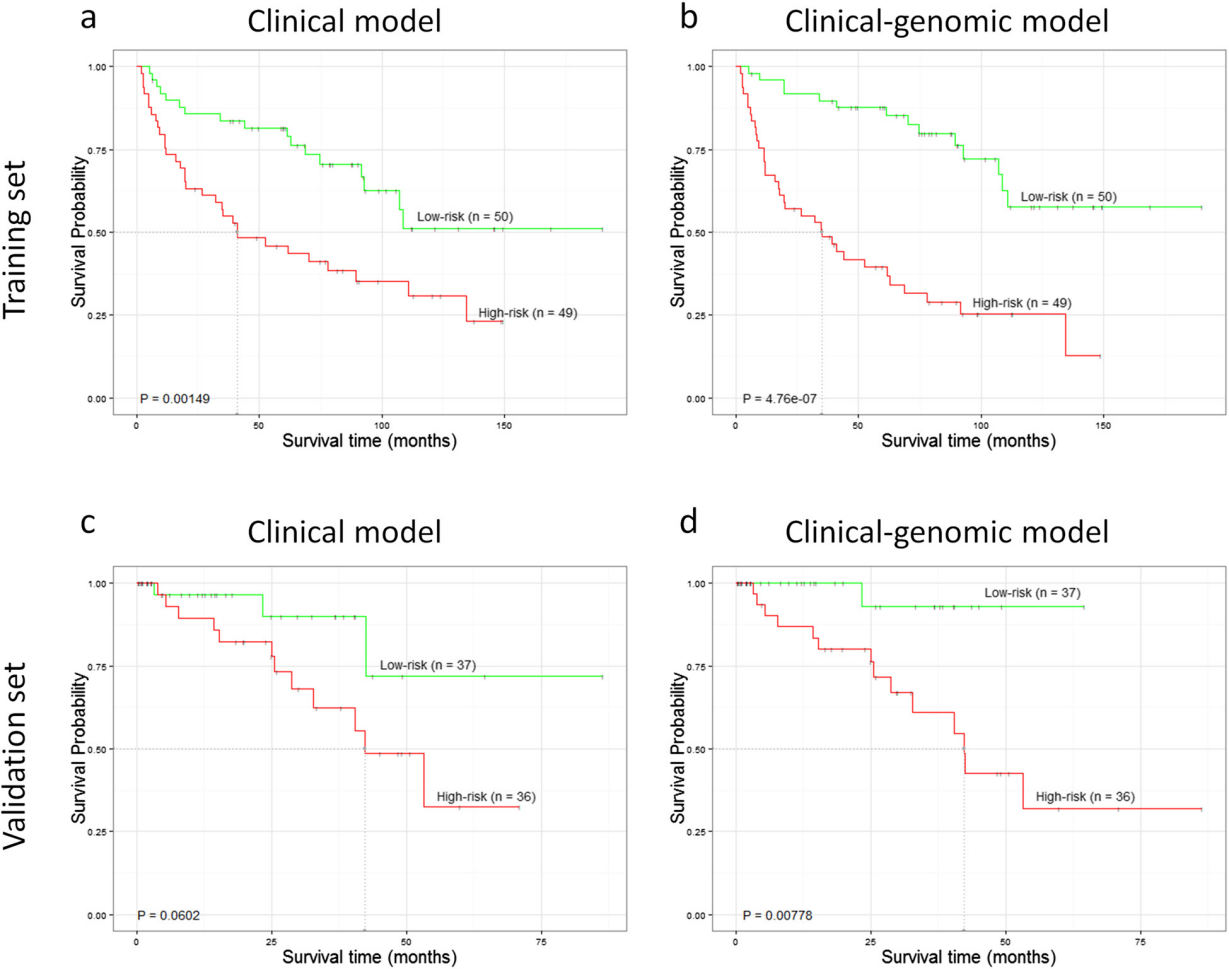
Kaplan Meier curves for the training (**a**, **b**) and validation (**c**, **d**) sets of ADC patients. For each case, patients were divided into two risk groups according to the predicted risk using either clinical (**a**, **c**) or clinical-genomic data (**b**, **d**). Survival curves were compared using log-rank test p-values

**Fig. 3:**
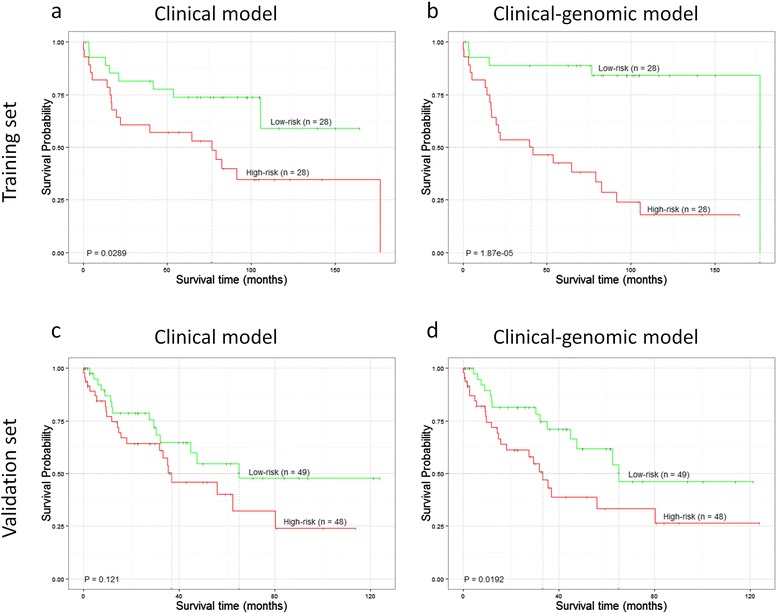
Kaplan Meier curves for the training (**a**, **b**) and validation (**c**, **d**) sets of SCC patients. For each case, patients were divided into two risk groups according to the predicted risk using either clinical (**a**, **c**) or clinical-genomic data (**b**, **d**). Survival curves were compared using log-rank test *p*-values

The prognostic role of the clinical-genomic signatures was evaluated in the training set. Risk scores were calculated according to the clinical-genomic signatures and dichotomized with the medians of the scores (therefore, in each histological subtype, the low and high risk groups included the same number of patients). Furthermore, the prognostic capacity of the signatures was validated in two independent datasets, one for each histological subtype. All prognostic significances of both the clinical-genomic and the clinical models are shown in Table 2 and Figs. 2 and 3. The clinical-genomic signatures outperformed the clinical signatures in both the training and validation sets, i.e. the p-values were smaller for the clinical-genomic models than for the clinical models and a wider separation of the Kaplan-Meier survival curves was clearly observed.

**Table 2:** Prognostic evaluation of the clinical-genomic and clinical signatures among the ADC and SCC patients in the corresponding training and validation sets

Datasets	Subtype	Type of signature	HR (95 % CI)	*p*-value^*^	*p*-value^**^
Training sets	ADC	Clinical-genomic	2.63 (1.95–3.53)	8.377e–11	4.76e–7
		Clinical	2.72 (1.62–4.55)	7.064e–5	0.0015
	SCC	Clinical-genomic	4.06 (2.20–7.46)	3.176e–6	1.87e–5
		Clinical	2.72 (1.36–5.45)	0.002	0.029
Validation sets	ADC	Clinical-genomic	2.1 (1.12–3.93)	0.01	0.008
		Clinical	2.09 (0.86–5.06)	0.05	0.06
	SCC	Clinical-genomic	1.56 (1.10–2.24)	0.007	0.019
		Clinical	1.42 (0.89–2.25)	0.07	0.121

^*^One-tailed p-value using the Cox proportional hazard model

^**^Log-rank test p-value

In order to elucidate whether the differences between the clinical and the clinical-genomic models were statistically significant, we performed the Harrell’s comparison test [28], separately for ADC and SCC. This test can be used to evaluate if a prediction model significantly outperforms a reference model. The Harrell’s test *p*-values were computed between clinical-genomic and clinical or genetic predictors for both ADC and SCC. Table 3 shows that the clinical-genomic signatures significantly outperformed the clinical signatures as prognostic models in both the training and validation sets, except for the ADC signature in the validation set, which despite having a better performance (*p*-value below 0.5) did not reach statistical significance. Nevertheless, compared to clinical-genomic signatures, the genetic signatures for both ADC and SCC validation sets (not included in Table 3) had a worse performance (*P* = 0.066 for ADC and *P* = 0.020 for SCC).

**Table 3:** Statistical comparison between clinical-genomic and clinical prognostic models. *p-values from the Harrell’s test

Dataset	Subtypes	*p*-values*
Training set	ADC	1.4601e–10
	SCC	3.3228e–09
Validation set	ADC	0.134
	SCC	0.0005

## Discussion

In this study, we have developed two clinical-genomic signatures to predict OS in chemotherapy-naïve, early stage ADC and SCC lung cancer patients. These signatures were based on the combination of clinical data and copy number alterations of a limited number of genes, and were validated in independent series.

To date, most of the published prognostic profiles are gene expression profiles based on RNA levels [5–7]. In our view, our prognostic signature based on CNA may have stronger prospects of clinical utility due to the higher stability of DNA when compared to RNA. In relation to other CNA survival predictors in the literature, most of them only inform about the prognostic value of individual gene or chomosomal region alterations [14, 15]. In our case, the group of genes selected for our profiles provides a more precise correlation with outcomes than individual genes. Moreover, in our selection algorithm, we only included those genes with a positive correlation between CNA and gene expression.

One of the challenges for this type of studies is the sample size required to achieve sufficient statistical robustness. This is especially a problem when early stage lung cancer patients are studied, as the numbers of events (disease progression or cancer-related death) are low. However, the availability of previously published array data allowed us to overcome this difficulty, combining DNA copy number data from other studies. In order to include comparable tumor samples, we minimized intra-tumor heterogeneity by performing an *in silico* tumor purity correction step for each of the samples, an approach that has been developed recently [29]. Still, since the number of samples is much smaller than the number of analyzed genes, a proper selection of the genes included in the signature is more important than the algorithm adopted to generate the survival model. With the aim to ease the feature selection process, we selected only those genes with prognostic value based on two external databases, (Prognoscan and GeneSigDB) and significant correlation between CNA and gene expression. This comprehensive selection process allowed us to derive two profiles that included a reduced number of genes (5 genes for the SCC profile and 7 genes for the ADC profile). A low number of genes in the final signature increases the feasibility of the clinical application of these signatures.

Genomic-based prognostic signatures usually include only genetic aberrations without taking into account well-established clinical prognostic features such as age and stage. Here, we proposed an integrated clinical-genomic signature. The clinical-genomic profile outperformed both the clinical-only data (see Figs. 2 and 3) or genetic-only data (see Additional file 6: Figures S9 and S10).

An additional novelty of our study is the description of a prognostic profile for SCC lung cancer patients. Most of the lung cancer prognostic signatures have been proposed for ADC and a prognostic signature for SCC is still missing. Although copy number and expression profiles of SCC lung carcinomas have been extensively described [10, 30, 31], assigning these patients into groups of different prognosis is still a challenge [13], maybe due to the potential existence of several biological subtypes within the SCC category [13, 32]. In our study the performance of the clinical-genomic model in SCC was lower compared to the ADC series, yet the clinical-genomic approach, was still more predictive compared to the clinical model.

According to the ADC clinical-genomic profile, patients with a higher risk of death showed an increase in the copy number of *YES1, TYMS, MYO1E, SLC25A20* and *PSMA4*, and a decrease in the copy number of *HMGN1* and *POFUT2. YES1* is a non-receptor tyrosine kinase from the SRC family kinase proteins. Previous studies in different neoplasms have shown that *in vitro* knock down of YES1 expression induce cell growth and metastasis reduction [33]. *TYMS* has been extensively studied in lung cancer as a prognostic marker of survival and a predictive marker of response to pemetrexed [34] and 5-FU. *HMGN1* protein binds to nucleosome and modifies chromatin structure. It participates in the repairing process of DNA lesions following UV light exposure and ionizing irradiation. In addition, *HMGN1* controls the transcription process of some oncogenes and tumor suppressor genes involved in tumor progression, mainly suppressing the development of cancer [35]. Expression of *PSMA4* is up-regulated in lung cancer [36]. It has been related to lung cancer proliferation and apoptosis and it is one of the genes located in the 15q24–25.1 region associated with lung cancer risk in western populations [37]. *MYO1E* codes for the class I myosin, involved in receptor mediated endocytosis [38]. Increased levels of *MYO1E* mRNA have been associated with recurrence of hepatocellular carcinoma [39]. *POFUT2* is an O-fucosyltransferase responsible for the O-fucosylation of thrombospondin type 1 and EGF repeats [40]. There is limited evidence on the role of this protein in cancer, but results of Pofut2 knockout mice showed that the loss of the protein leads to epithelial-mesenchymal transition in mouse embryogenesis, suggesting an important role of the protein in cancer [41]. No studies have been performed in cancer to investigate copy number or gene expression alterations of *SLC25A20,* a transport protein present in the mitochondrial membrane.

According to the SCC clinical-genomic profile, an increase in the copy number of *ZNF292* and *DICER1,* and a decrease in the copy number of *TRA2B,GPD1L*, and *CTNND1* is an indicator of poor prognosis. The association found in our study between CNA of *GPD1L* and *CTNND1* and prognosis is consistent with the associations previously described not only in lung cancer but also in other neoplasms [42, 43]. *TRA2B* amplification has been described in several neoplasms, including lung cancer [44]. Upregulation of Tra2β protein has been associated with aggressiveness in cervical cancer [45]; however the role of this protein in lung cancer is unknown. No association between *ZNF292* expression and progression has been described, and contrasting results have been published regarding the prognostic value of the *DICER1* abnormal expression, depending on the tumor origin. Whereas low *DICER1* expression has been correlated with worse prognosis in chronic lymphocytic leukemia and melanoma [46, 47], high expression has been correlated with reduced survival in prostate adenocarcinomas and colorectal carcinomas [48, 49]. In our clinical-genomic profile, *DICER1* gene deletions were associated with low risk in SCC patients. Previous studies in lung cancer showed association between low level expression and poor survival [50] in adenocarcinoma samples or in series where ADC and SCC were analyzed together. Further studies are needed to clarify if aberrant *DICER1* expression has a different prognostic role in SCC and ADC subtypes or to explain the apparently opposite direction of the prognostic value of *DICER1* gene copy number and the expression of the transcribed protein.

Our prognostic profiles were validated in silico in two independent series of stage I and II ADC and SCC samples from TCGA. However, in order to apply these profiles in the clinic, future validations using routinely available techniques for CNA analysis, such as FISH or qPCR, as well as independent prospective cohorts are needed.

## Conclusion

In conclusion, based on our novel selection algorithm, we have designed two prognostic profiles for stage I and II lung ADC and SCC patients based on both CNA and clinical features. These combined clinical-genomic profiles were able to improve the prognostic classification of patients based on clinical characteristics. After a prospective validation, this new tool could guide clinical management in early-stage lung cancer patients.

## Additional files

Additional file 1:
**Supplementary Methods, Results, Figure Legends & Tables.** (DOCX 94 kb) Additional file 2:
**Preselected 513 candidate genes for ADC.** (XLSX 61 kb) Additional file 3:
**Preselected 150 candidate genes for SCC.** (XLSX 24 kb) Additional file 4:
**GO biological processes highly significantly enriched in the 513 preselected gene set for ADC.** (XLSX 19 kb) Additional file 5:
**GO biological processes highly significantly enriched in the 150 preselected gene set for SCC.** (XLSX 9 kb) Additional file 6:
**Kaplan Meier curves for the validation set of ADC (Figure S9) and SCC (Figure S10) using the genomic model (A) and the clinical-genomic model (B) highlighted in red. Patients were divided into two risk groups according to the predicted risk.** Survival curves were compared using the log-rank test p-values. (PPTX 6554 kb)

## Abbreviations

NSCLC: Non-small cell lung cancer
ADC: Adenocarcinoma
SCC: Squamous cell carcinoma
ACT: Adjuvant Chemotherapy
CNA: Copy number aberration
TCGA: The Cancer Genome Atlas
MDA: MD Anderson
CIMA: Center for Applied Medical Research
CUN: Clinica Universidad de Navarra
HUMV: Marques de Valdecilla University Hospital
AIC: Akaike Information Criterion
OS: Overall Survival
