# Supplementary material for: Combined clinical and genomic signatures for the prognosis of early stage non-small cell lung cancer based on gene copy number alterations
Source: BMC Genomics. 2015 Oct 6;16:752. doi: 10.1186/s12864-015-1935-0 (PMC4595201; doi:10.1186/s12864-015-1935-0)
Supplement: Additional file 1: — Supplementary Methods, Results, Figure Legends & Tables. (DOCX 94 kb) [file 12864_2015_1935_MOESM1_ESM.docx]

Supplementary Information

**Table of contents**

[Supplementary Methods 1](#_Toc413337601)

[*Data gathering* 1](#_Toc413337602)

[*Core algorithm* 1](#_Toc413337603)

[Supplementary Results 7](#_Toc413337604)

[*Survival model inference* 7](#_Toc413337605)

[*Comparison of gene-specific copy number between high-risk and low-risk signatures* 8](#_Toc413337606)

[Supplementary References 10](#_Toc413337607)

[Supplementary Figure Legends 12](#_Toc413337608)

[Supplementary Tables 14](#_Toc413337609)

# Supplementary Methods

## *Data gathering*

Supplementary Tables S1 and S2 summarize the clinical characteristics and the type of genetic study performed in each series of ADC and SCC patients.

## *Core algorithm*

In this section, the most critical steps of the core algorithm (See Supplementary Figure S1) are described.

1. **Tumor purity correction**

The tumor purity for each sample was estimated via the GPHMM algorithm[1]. Then, a copy number adaptation was performed. For this, the following linear relationship was stated:

| $TCN_{measured,ij}=\beta_{j}\cdot TCN_{tumor,ij}+\left(1-\beta_{j}\right)\cdot TCN_{normal,ij}$ | (1) |
| --- | --- |

where $TCN_{measured,ij}$ indicates the total copy number measured from the summarized signals for SNP "i" and sample "j"; $TCN_{tumor,ij}$ indicates the total copy number from pure tumor tissue for SNP "i" and sample "j"; $TCN_{normal,ij}$ indicates the total copy number from pure normal tissue for SNP "i" and sample "j" and $\beta_{j}$ indicates the proportion of tumor cells from the analyzed tissue sample from sample "j". The latter is also known as tumor purity.

In this study, autosomes (i.e. chromosomes from 1 to 22) were only considered. Since total copy number was assumed to be constant along the autosomal regions and equal to 2 copies in normal tissue $\left(\text{i.e. }TCN_{normal,ij}=2\right)$, the total copy number values for the pure tumor case were estimated as:

| $TCN_{tumor,ij}=\frac{TCN_{measured,ij}-\left(1-\beta_{j}\right)\cdot2}{\beta_{j}}$ | (2) |
| --- | --- |

This correction also reduces variation among samples both within the same dataset, and across datasets.

1. **Summarization of segmented copy number to gene copy number**

Frequently, several copy number (log2ratio) segments fall within the same gene for a given sample. To solve this problem, we assigned a single value to each gene. For this, a weighted median [2] was performed to the mean copy number values of the segments within the analyzed gene, where the weights correspond to the length of the corresponding segments within the gene.

1. **Gene Filtering**

Survival analysis included genes that: a) presented a significant positive correlation (local FDR adjusted q-value<0.2) between gene copy number and gene expression profiles, and b) their expression profiles correlated with overall survival (OS).

The correlation between gene copy number and gene expression profiles was computed for all the analyzed datasets with both types of genetic data.

The correlative association between gene expression profiles and survival outcome was measured based on two external genomic databases: GeneSigDB[3] and Prognoscan[4].

*GeneSigDB*

A first candidate gene list was downloaded from GeneSigDB. This list was generated from curated gene signatures related to patient survival for each NSCLC subtype (See Supplementary Table S3).

*Prognoscan*

A second list was generated based on Prognoscan data. In this case, a meta-analysis for each gene was performed across all the available datasets on Prognoscan database, in order to determine the most significant genes that predicted OS for both ADC and SCC, separately.

In Prognoscan database, the number of datasets available for each gene varied from 7 to 8 and 1 to 2 datasets for ADC and SCC, respectively. In SCC, since the available number of datasets was very low, the filter provided by Prognoscan was not considered.

In the first step, we focused on the two-tailed Cox p-values of OS for each gene (See Supplementary Figure S2). Then, we transformed these two-tailed p-values to one-tailed p-values with the aid of the corresponding hazard ratios. Since the one-tailed Cox p-values were probe-specific of the analyzed gene, a global Cox p-value of the gene was computed for each dataset. Specifically, the Irwin hall distribution was used to obtain the combined Cox p-value of the analyzed gene for each dataset separately.

Once the gene-specific Cox p-value for each dataset was obtained, we summarized these p-values by datasets using the Stouffer's method. This approach is closely related to Fisher's method. Nevertheless, it is based on Z-scores rather than on p-values. Since the Z-score for the i^th^ dataset is defined as $Z_{i}=\varphi^{-1}\left(1-p_{i}\right)$, where *Φ* is the standard normal cumulative distribution function, the Z-score for the overall meta-analysis of the analyzed gene (Z') based on the Stouffer's methods is obtained as,

| $Z'=\frac{\sum_{i=1}^{k} Z_{i}}{\sqrt{k}}$ | (3) |
| --- | --- |

where $Z_{i}$ refers to the Z-score from the i^th^ dataset and k to the total number of datasets involved in the analyzed gene. One advantage of this approach is that it is straightforward to introduce weights. Therefore, if the i^th^ Z-score is weighted by $\omega_{i}$, then the corresponding overall meta-analysis Z-score (Z'') is defined as:

| $Z''=\frac{\sum_{i=1}^{k} \omega_{i}\cdot Z_{i}}{\sqrt{\sum_{i=1}^{k} \omega_{i}^{2}}}$ | (4) |
| --- | --- |

which follows a standard normal distribution under the null hypothesis. In this case, each weight $\omega_{i}$ refers to the square root of the sample size of the i^th^ dataset. Therefore, we computed the summarized Cox p-value for each gene from the weighted averaged Z-score provided by the Stouffer's method. Then, we corrected these summarized gene Cox p-values based on the local FDR approach [5–7]. Using an adjusted q-value < 0.2 as the cut-off, we obtained the gene list provided by Prognoscan.

The final list of genes, based on both external genomic databases, was the union of both lists. The filtering processes and database search were performed separately for NSCLC subtypes. The candidate genes were therefore those with positive correlation between copy number and gene expression, and appear in any of the survival lists from GeneSigDB or Prognoscan.

1. **NetRank and Model Averaging**

The NetRank algorithm mimics the PageRank algorithm that Google uses to rank its results. Each page (gene in this case) has a relevance derived by the terms included in the search. Using the network of hyperlinks between pages and the relative importance of each page, the initial ranking generated by the relevance is changed to accommodate the additional information of the network. There is a tuning parameter (the damping factor) that modulates how much the network information alters the initial ranking. In our case the role of the hyperlink network is played by the positive correlation in the copy number profiles of the genes for the unlabeled samples and the role of the relevance is played by the statistical significance of each gene in the OS analysis using only the training set. The application of this algorithm as a prognostic method requires selecting both the damping factor and the number of genes to include in the model. These parameters were selected via the Akaike Information Criterion (AIC). The procedure to select these values is as follows: a grid of damping factors (from 0 to 0.9) and number of genes to include in the model (from 1 to 10) was performed. For each case of this grid of values, a Cox Proportional Hazard Model was evaluated. The final model is an weighted average of the best candidate models according to its AIC[8].

The AIC is a way of selecting a model from a set of models. This criterion looks for a model that has a good fit to the truth but with few parameters. AIC is defined as:

| $AIC=-2\cdot\log(L)+2\cdot K$ | (5) |
| --- | --- |

where L, the likelihood, is the probability of the data given a model and K is the number of free parameters in the model. When there are several models, AIC_m_ scores can be also shown as ∆AIC_m_ scores. The latter scores are defined as the difference between the AIC values of each model “m” and the best model (with minimum AIC). Then, the relative likelihood (RL) of model “m” is defined as:

| $RL_{m}=e^{-\frac{1}{2}\cdot\left(AIC_{m}-AIC_{\min}\right)}=e^{-\frac{1}{2}\cdot\Delta AIC_{m}}$ | (6) |
| --- | --- |

Finally, the Akaike weights (AW) provide another measure of the strength of evidence for each model, and represent the ratio of ∆AIC_m_ values for each model "m" relative to the whole set of R candidate models.

| $AW_{m}=\frac{e^{-\frac{1}{2}\cdot\left(AIC_{m}-AIC_{\min}\right)}}{\sum_{r=1}^{R} e^{-\frac{1}{2}\cdot\left(AIC_{m}-AIC_{\min}\right)}}=\frac{e^{-\frac{1}{2}\cdot\Delta AIC_{m}}}{\sum_{r=1}^{R} e^{-\frac{1}{2}\cdot\Delta AIC_{r}}}$ | (7) |
| --- | --- |

Additionally, AW can be used for model averaging. Thus, in this study, the AW were used to generate the best weighted averaged model of the candidate models (∆AIC<2) based on the Akaike Information Criterion. This model averaging method is also known as smoothed AIC (SAIC).

# Supplementary Results

## *Survival model inference*

*Adenocarcinoma (ADC)*

The AIC values for the ADC training set were calculated for each pair of values for the number of genes selected (n) and the damping factor (d) (See Supplementary Figure S3a). The damping factor is related with the importance given to the CN-CN gene network. This parameter goes from 0 to 1, where d=0 indicates no influence and d=1 indicates full influence of the network. The number of genes was considered up to 10 genes. Notice that in this subfigure, a gray scale is used to represent the AIC values. Specifically, the higher the AIC value the lighter the gray tone is used. In order to determine the final gene signature, we selected the most competitive models (∆AIC<2) which are shown in dark gray in Supplementary Figure S3b. The obtained clinical-genomic model for ADC in the training phase contained 7 prognostic genes (See Supplementary Table S4).

*Squamous-cell carcinoma (SCC)*

Analogously, the AIC values for the SCC training set were calculated for each pair of values for the number of genes selected (n) and the damping factor (d) (See Supplementary Figure S4a). Again, in order to select the final gene signature, we selected the most competitive models (∆AIC<2) which are shown in dark gray in Supplementary Figure S4b. The obtained clinical-genomic model for SCC in the training phase contained 5 prognostic genes (See Supplementary Table S5).

## *Comparison of gene-specific copy number between high-risk and low-risk signatures*

*Adenocarcinoma (ADC)*

The influence of gene-specific copy number (log2ratio) aberrations on overall survival (OS) was analyzed. In Supplementary Figure S5, the difference in log2ratio between the high risk and low risk groups for each gene of the ADC 7-gene predictor is shown among the ADC samples of the training set. Wilcoxon test was used to assess differences in log2ratio between risk groups. In each subfigure, the Y-axis represents the copy number values (in log2ratio) and the X-axis indicates the high risk and low risk groups. The hazard ratio for each gene previously calculated (using the multivariate Cox regression) in the training set is coherent with the obtained gene-specific log2ratio profiles among both survival groups. Therefore, a subset of 5 genes (*YES1*, *TYMS*, *PSMA4*, *MYOE1* and *SLC25A20*), and another subset of 2 genes (*HMGN1* and *POFUT2*) were identified to present a risky and protective behavior, respectively. In addition, it can be observed that the gene ranking is different using multivariate Cox p-values compared with Wilcoxon test p-values. Notably, however for the multivariate Cox test, each gene was treated separately in combination with the clinical data, whereas for the Wilcoxon test, the survival risk groups were stratified according to the estimated survival risk scores based on the full clinical-genomic model. Therefore, for the latter, the influence of each gene in the final survival model is represented. A smaller p-value indicates a stronger influence of the gene in the survival model. It can be observed that all the genes were statistically significant (p-values < 0.05).

Supplementary Figure S6 shows the same approach applied to the validation set of ADC. The difference in log2ratio between low risk and high risk groups for each gene of the ADC prognostic signature in the validation set was coherent in significance and in direction if compared with the difference in log2ratio obtained for the training set. However, statistical significance was not achieved for the following genes: *HMGN1*, *POFUT2* and *SLC25A20*.

*Squamous-cell carcinoma (SCC)*

In Supplementary Figure S7, the same approach was applied to SCC. The difference in log2ratio between risk groups for each gene of the SCC 5-gene predictor is shown among the SCC samples of the training set. Again, in the training set, the hazard ratio for each gene was coherent with the obtained log2ratio profiles. In contrast to ADC, some genes clearly present copy number offsets. For example, *TRA2B* and *GPD1L* have a positive and negative offset in log2ratio, respectively. Indeed, *TRA2B* is amplified in all samples, whereas *GPD1L* is deleted. Again, notice that the gene ranking is different using the multivariate Cox p-values if compared with the Wilcoxon test p-values. *TRA2B* and *GPD1L* were not statistically significant using the Wilcoxon test.

Supplementary Figure S8 shows the same approach applied to the validation set of SCC. In contrast to ADC, the differences in log2ratio between low risk and high risk groups for the validation set were not so coherent with the differences obtained for the training set. Even though *TRA2B* and *CTNND1* were coherent in direction, only *TRA2B* was statistically significant (Wilcoxon p-value < 0.05).

# Supplementary References

1. Li A, Liu Z, Lezon-Geyda K, Sarkar S, Lannin D, Schulz V, Krop I, Winer E, Harris L, Tuck D: **GPHMM: an integrated hidden Markov model for identification of copy number alteration and loss of heterozygosity in complex tumor samples using whole genome SNP arrays.** *Nucleic Acids Res* 2011, **39**:4928–41.

2. Cormen TT, Leiserson CE, Rivest RL: *Introduction to Algorithms*. Cambridge, MA, USA: MIT Press; 1990.

3. Culhane AC, Schröder MS, Sultana R, Picard SC, Martinelli EN, Kelly C, Haibe-Kains B, Kapushesky M, St Pierre A-A, Flahive W, Picard KC, Gusenleitner D, Papenhausen G, O’Connor N, Correll M, Quackenbush J: **GeneSigDB: a manually curated database and resource for analysis of gene expression signatures.** *Nucleic Acids Res* 2012, **40**(Database issue):D1060–6.

4. Mizuno H, Kitada K, Nakai K, Sarai A: **PrognoScan: a new database for meta-analysis of the prognostic value of genes.** *BMC Med Genomics* 2009, **2**:18.

5. Efron B: **Large-scale simultaneous hypothesis testing**. *J Am Stat Assoc* 2004, **99**.

6. Efron B: **Size, power and false discovery rates**. *Ann Stat* 2007, **35**:1351–1377.

7. Efron B: **Correlation and large-scale simultaneous significance testing**. *J Am Stat Assoc* 2007, **102**.

8. Burnham KP, Anderson DR: *Model Selection and Multi-Model Inference: A Practical Information-Theoretic Approach*. Springer; 2002.

9. Gordon GJ, Richards WG, Sugarbaker DJ, Jaklitsch MT, Bueno R: **A prognostic test for adenocarcinoma of the lung from gene expression profiling data.** *Cancer Epidemiol Biomarkers Prev* 2003, **12**:905–10.

10. Khodarev NN, Pitroda SP, Beckett M a, MacDermed DM, Huang L, Kufe DW, Weichselbaum RR: **MUC1-induced transcriptional programs associated with tumorigenesis predict outcome in breast and lung cancer.** *Cancer Res* 2009, **69**:2833–7.

11. Kikuchi T, Daigo Y, Katagiri T, Tsunoda T, Okada K, Kakiuchi S, Zembutsu H, Furukawa Y, Kawamura M, Kobayashi K, Imai K, Nakamura Y: **Expression profiles of non-small cell lung cancers on cDNA microarrays: identification of genes for prediction of lymph-node metastasis and sensitivity to anti-cancer drugs.** *Oncogene* 2003, **22**:2192–205.

12. Larsen JE, Pavey SJ, Passmore LH, Bowman R V, Hayward NK, Fong KM: **Gene expression signature predicts recurrence in lung adenocarcinoma.** *Clin Cancer Res* 2007, **13**:2946–54.

13. Miura K, Bowman ED, Simon R, Peng AC, Robles AI, Jones RT, Katagiri T, He P, Mizukami H, Charboneau L, Kikuchi T, Liotta L a, Nakamura Y, Harris CC: **Laser capture microdissection and microarray expression analysis of lung adenocarcinoma reveals tobacco smoking- and prognosis-related molecular profiles.** *Cancer Res* 2002, **62**:3244–50.

14. Moran CJ, Arenberg D a, Huang C-C, Giordano TJ, Thomas DG, Misek DE, Chen G, Iannettoni MD, Orringer MB, Hanash S, Beer DG: **RANTES expression is a predictor of survival in stage I lung adenocarcinoma.** *Clin Cancer Res* 2002, **8**:3803–12.

15. Larsen JE, Pavey SJ, Passmore LH, Bowman R, Clarke BE, Hayward NK, Fong KM: **Expression profiling defines a recurrence signature in lung squamous cell carcinoma.** *Carcinogenesis* 2007, **28**:760–6.

16. Sun Z, Yang P, Aubry M-C, Kosari F, Endo C, Molina J, Vasmatzis G: **Can gene expression profiling predict survival for patients with squamous cell carcinoma of the lung?** *Mol Cancer* 2004, **3**:35.

17. Tomida S, Koshikawa K, Yatabe Y, Harano T, Ogura N, Mitsudomi T, Some M, Yanagisawa K, Takahashi T, Osada H, Takahashi T: **Gene expression-based, individualized outcome prediction for surgically treated lung cancer patients.** *Oncogene* 2004, **23**:5360–70.

# Supplementary Figure Legends

Supplementary Figure S1. Panel 1, the main processing pipeline steps. Panel 2, the model selection pipeline followed to achieve the final clinical-genomic signature. The four most critical steps are highlighted in rectangle boxes. *Gene Expression (GE). **Databases (DDBB).

Supplementary Figure S2. Prognostic value of TYMS for each available dataset in Prognoscan database in ADC OS.

Supplementary Figure S3. a) AIC values for the ADC training set for each pair of values for the number of genes selected (n) and the damping factor (d). A gray scale is used to represent the AIC values. Specifically, the higher the AIC value the lighter the gray tone is used. b), the most competitive models (∆AIC<2) are shown in dark gray.

Supplementary Figure S4. a), AIC values for the SCC training set are shown for each pair of values for the number of genes selected (n) and the damping factor (d). A gray scale is used to represent the AIC values. Specifically, the higher the AIC value the lighter the gray tone is used. In b), the most competitive models (∆AIC<2) are shown in dark gray.

Supplementary Figure S5. Gene log2ratio differences between low-risk and high-risk groups are shown for the ADC training set. Wilcoxon test was used to assess differences in log2ratio between risk groups. The shown p-values are two-tailed. The Y-axis represents the copy number data (in log2ratio) and the X-axis represents the risk groups.

Supplementary Figure S6. Gene log2ratio differences between low-risk and high-risk groups are shown for the ADC validation set. The interpretation is equivalent to Supplementary Figure S5. The genes shown constitute the ADC gene signature.

Supplementary Figure S7. Gene log2ratio differences between low-risk and high-risk groups are shown for the SCC training set. The interpretation is equivalent to Supplementary Figure S5. The genes shown constitute the SCC gene signature (Notice the change in the log2ratio scale for TRA2B).

Supplementary Figure S8. Gene log2ratio differences between low-risk and high-risk groups are shown for the SCC validation set. The interpretation is equivalent to Supplementary Figure S5. The genes shown constitute the SCC gene signature (Notice the change in the log2ratio scale for TRA2B).

# Supplementary Tables

**Supplementary Table S1.** Clinical data and molecular platforms used for the ADC training and validation cohorts.

|  | | CIMA-CUN-HUMV | MDA | GSE28582 | GSE25016 | GSE34140 | TCGA (Validation) |
| --- | --- | --- | --- | --- | --- | --- | --- |
| Number of samples | | 16 | 50 | 33 | 77 | 162 | 73 |
| Sex | Male | 11(68.75%) | 20(40.00%) | 12(36.36%) | NA | NA | 31(42.47%) |
|  | Female | 5(31.25%) | 30(60.00%) | 21(63.64%) | NA | NA | 42(57.53%) |
| Age | ≤65 years | 8(50.00%) | 23(46.00%) | 15(45.45%) | NA | NA | 32(43.84%) |
|  | >65 years | 8(50.00%) | 27(54.00%) | 18(54.55%) | NA | NA | 41(56.16%) |
| Clinical Stage | IA | 6(37.50%) | 16(32.00%) | 9(27.27%) | NA | 127(78.40%) | 22(30.14%) |
|  | IB | 10(62.50%) | 22(44.00%) | 18(54.55%) | NA |  | 34(46.58%) |
|  | IIA | NA | 5(10.00%) | 2(6.06%) | NA | 35(21.60%) | 3(4.11%) |
|  | IIB | NA | 7(14.00%) | 4(12.12%) | NA |  | 14(19.18%) |
| Survival data | Alive | 11(68.75%) | 32(64.00%) | 7(21.21%) | NA | NA | 58(79.45%) |
|  | Dead | 5(31.25%) | 18(36.00%) | 26(78.79%) | NA | NA | 15(20.55%) |
|  | Median follow-up (months) | 77 | 62 | 20 | NA | NA | 20 |
| Microarray platform | Copy Number | Affy 500K | Agilent 244K CGH | Affy 250K_Nsp | Affy GWS6.0 | Affy 250K_Nsp | Affy GWS6.0 |
|  | Gene Expression | NA | NA | Affy U133 Plus2 | NA | NA | Agilent G4502A |

*NA indicates Not Available data.

**Supplementary Table S2.** Clinical data and molecular platforms used for the SCC training and validation cohorts.

|  | | CIMA-CUN-HUMV | MDA | GSE28582 | GSE25016 | GSE34140 | TCGA (Validation) |
| --- | --- | --- | --- | --- | --- | --- | --- |
| Number of samples | | 23 | 14 | 19 | 155 | 83 | 97 |
| Sex | Male | 21(91.30%) | 9(64.29%) | 12(63.16%) | NA | NA | 66(68.04%) |
|  | Female | 2(8.70%) | 5(35.71%) | 7(36.84%) | NA | NA | 31(31.96%) |
| Age | ≤65 years | 10(43.48%) | 5(35.71%) | 6(31.58%) | NA | NA | 34(35.05%) |
|  | >65 years | 13(56.52%) | 9(64.29%) | 13(68.42%) | NA | NA | 63(64.95%) |
| Clinical Stage | IA | 9(39.13%) | 4(28.57%) | NA | NA | 71(85.54%) | 19(19.59%) |
|  | IB | 10(43.48%) | 7(50.00%) | 13(68.42%) | NA |  | 53(54.64%) |
|  | IIA | 4(17.39%) | 1(7.14%) | NA | NA | 12(14.46%) | 6(6.19%) |
|  | IIB | NA | 2(14.29%) | 6(31.58%) | NA |  | 19(19.59%) |
| Survival data | Alive | 19(82.61%) | 6(42.86%) | 5(26.32%) | NA | NA | 59(60.82%) |
|  | Dead | 4(17.39%) | 8(57.14%) | 14(73.68%) | NA | NA | 38(39.18%) |
|  | Median follow-up (months) | 76 | 81 | 20 | NA | NA | 23 |
| Microarray platform | Copy Number | Affy 500K | Agilent 244K CGH | Affy 250K_Nsp | Affy GWS6.0 | Affy 250K_Nsp | Affy GWS6.0 |
|  | Gene Expression | NA | NA | Affy U133 Plus2 | NA | NA | Agilent G4502A |

*NA indicates Not Available data.

Supplementary Table S3. GeneSigDB references used in the correlative association between gene expression profiles and survival outcome are shown.

| NSCLC Subtype | GeneSigDB References |
| --- | --- |
| ADC | [9–14] |
| SCC | [11, 15–17] |

**Supplementary Table S4.** Multivariate analysis for overall survival among patients with ADC in the training set.

| **Characteristic** | **Description** | **HR (95% CI)*** | **p-value**** |
| --- | --- | --- | --- |
| Age | Continuous age (in years) | 1.01 (0.98-1.04) | 0.540 |
| IB vs IA | Incremental risk IB relative to IA | 2.92 (1.34-6.33) | 0.007 |
| IIB vs IB | Incremental risk IIB relative to IB | 2.16 (0.99-4.74) | 0.054 |
| *YES1* | Yamaguchi sarcoma viral oncogenehomolog 1 | 5.62 (2.43-12.98) | <0.001 |
| *TYMS* | Thymidylatesynthase | 6.83 (2.63-17.75) | <0.001 |
| *HMGN1* | High mobility group nucleosome binding domain 1 | 0.22 (0.08-0.61) | 0.004 |
| *PSMA4* | Proteasome (prosome, macropain) subunit, alpha type, 4 | 4.14 (1.6-10.7) | 0.003 |
| *MYO1E* | Myosin IE | 5.67 (1.78-18.11) | 0.003 |
| *POFUT2* | Protein O-fucosyltransferase 2 | 0.36 (0.16-0.79) | 0.010 |
| *SLC25A20* | Solute carrier family 25 | 2.39 (0.88-6.47) | 0.088 |

*The hazard ratios associated with the clinical covariates (age and stages IB and IIB) were computed separately. The hazard ratio for each gene was estimated via a multivariate Cox regression that included the corresponding gene and the clinical factors.

**p-values are two-tailed.

**Supplementary Table S5.** Multivariate analysis for overall survival among patients with SCC in the training set.

| **Characteristic** | **Description** | **HR (95% CI)*** | **p-value**** |
| --- | --- | --- | --- |
| Age | Continuous age (in years) | 1.08 (1.02-1.15) | 0.008 |
| IB vs IA | Incremental risk IB relative to IA | 1.71 (0.57-5.10) | 0.340 |
| IIB vs IB | Incremental risk IIB relative to IB | 1.44 (0.47-4.36) | 0.520 |
| *TRA2B* | Transformer 2 beta homolog (Drosophila) | 0.33 (0.13-0.84) | 0.019 |
| *ZNF292* | Zinc finger protein 292 | 8.42 (1.28-55.21) | 0.026 |
| *CTNND1* | Catenin (cadherin-associated protein), delta 1 | 0.22 (0.06-0.85) | 0.028 |
| *GPD1L* | Glycerol-3-phosphate dehydrogenase 1-like | 0.15 (0.03-0.82) | 0.029 |
| *DICER1* | Dicer 1, ribonuclease type III | 2.86 (1.09-7.54) | 0.033 |

*The hazard ratios associated with the clinical covariates (age and stages IB and IIB) were computed separately. The hazard ratio for each gene was estimated via a multivariate Cox regression that included the corresponding gene and the clinical factors.

**p-values are two-tailed.
