# Supplementary material for: Combined clinical and genomic signatures for the prognosis of early stage non-small cell lung cancer based on gene copy number alterations
Source: BMC Genomics. 2015 Oct 6;16:752. doi: 10.1186/s12864-015-1935-0 (PMC4595201; doi:10.1186/s12864-015-1935-0)
Supplement: Additional file 6: — Kaplan Meier curves for the validation set of ADC (Figure S9) and SCC (Figure S10) using the genomic model (A) and the clinical-genomic model (B) highlighted in red. Patients were divided into two risk groups according to the predicted risk. Survival curves were compared using the log-rank test p-values. (PPTX 6554 kb) [file 12864_2015_1935_MOESM6_ESM.pptx]

## Slide 1
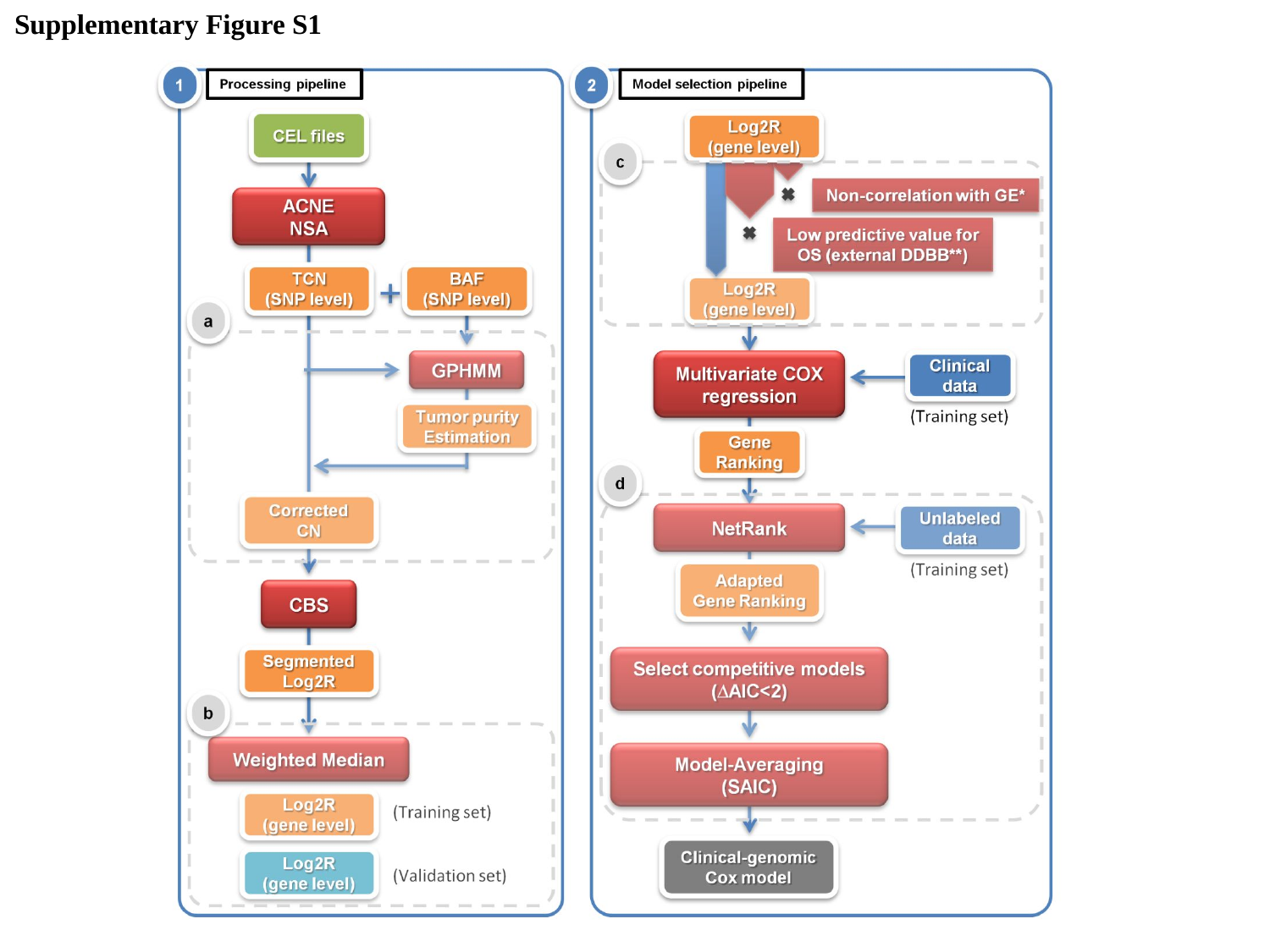

Supplementary Figure S1

## Slide 2
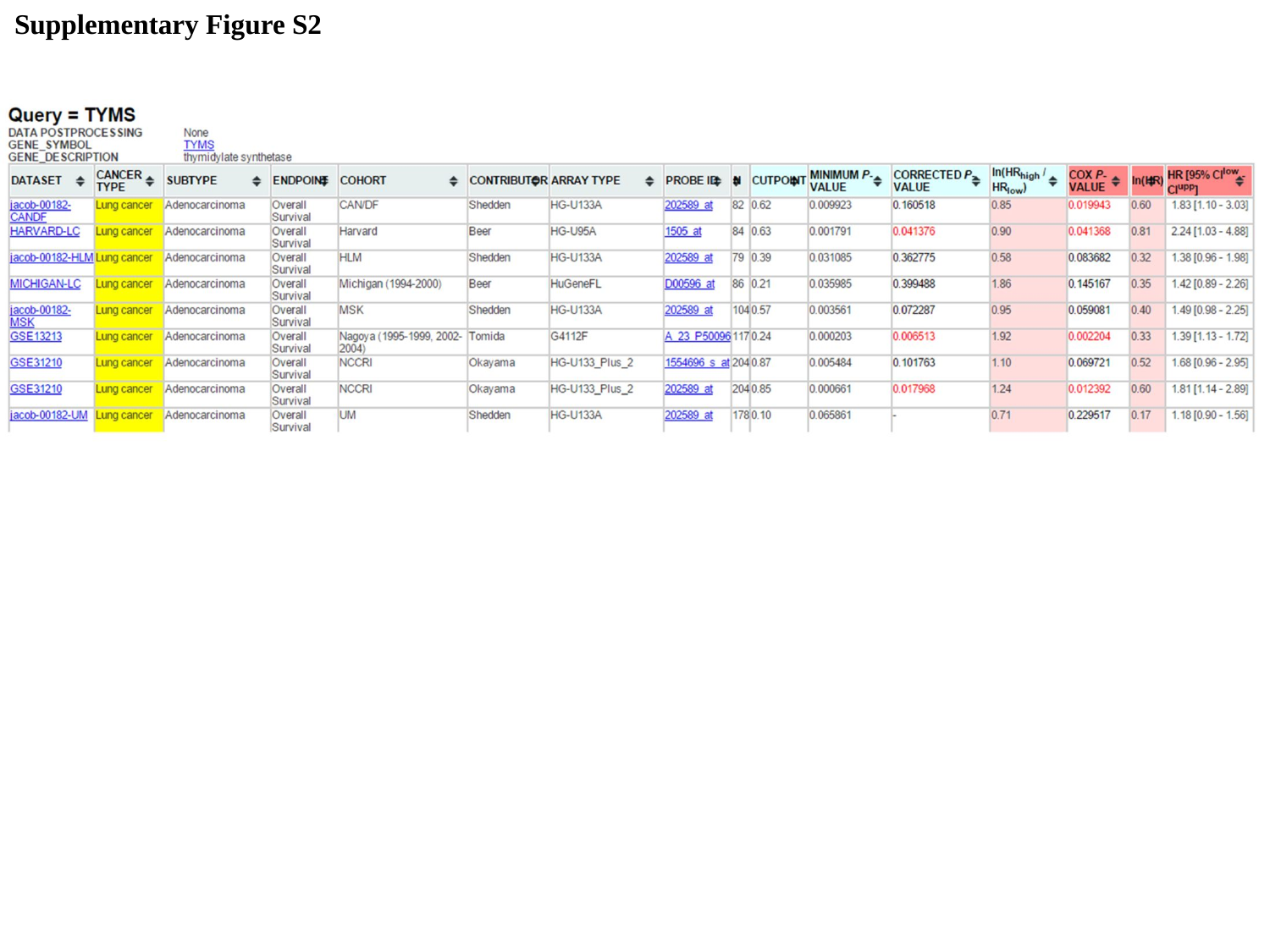

Supplementary Figure S2

## Slide 3
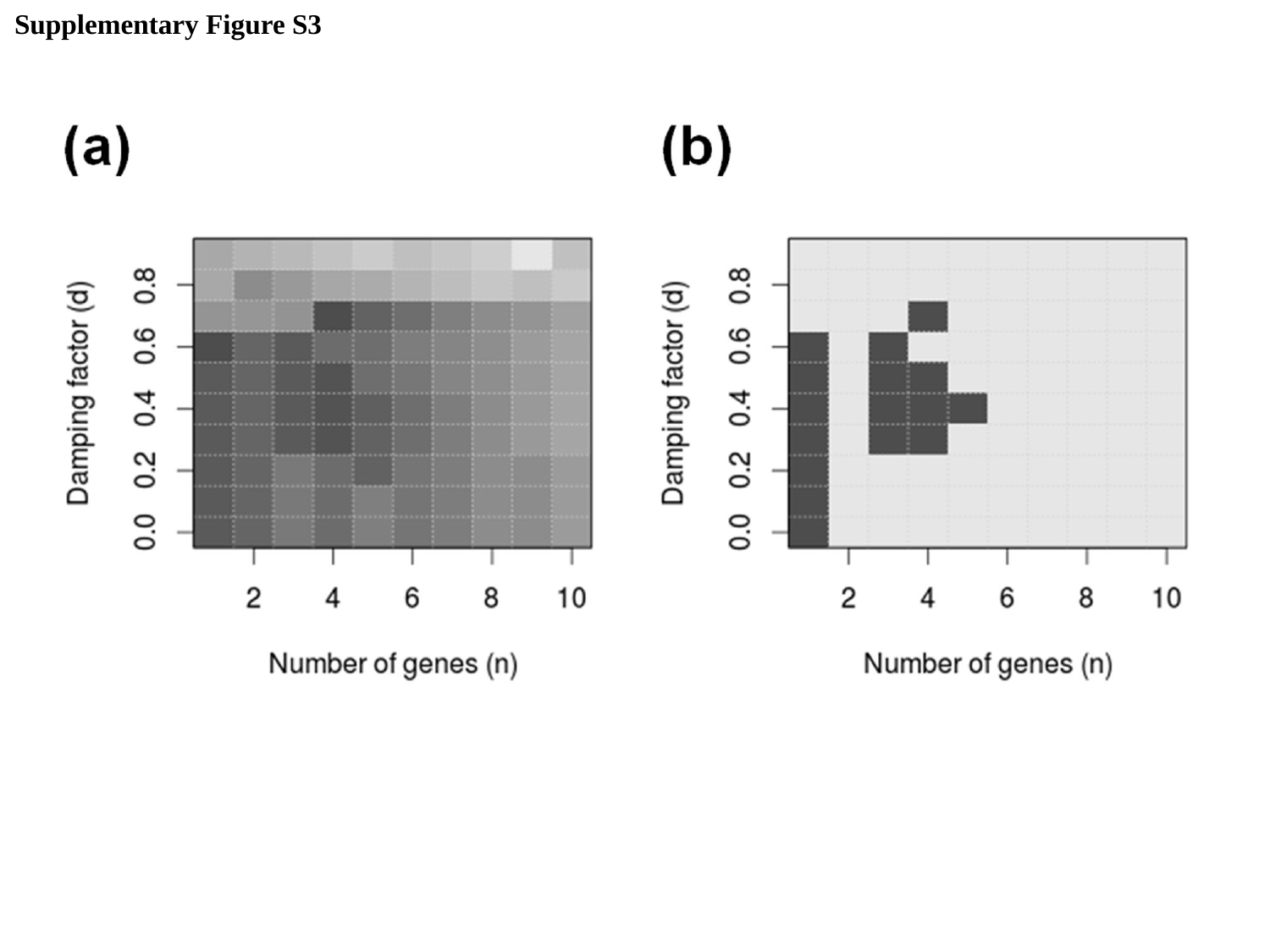

Supplementary Figure S3

## Slide 4
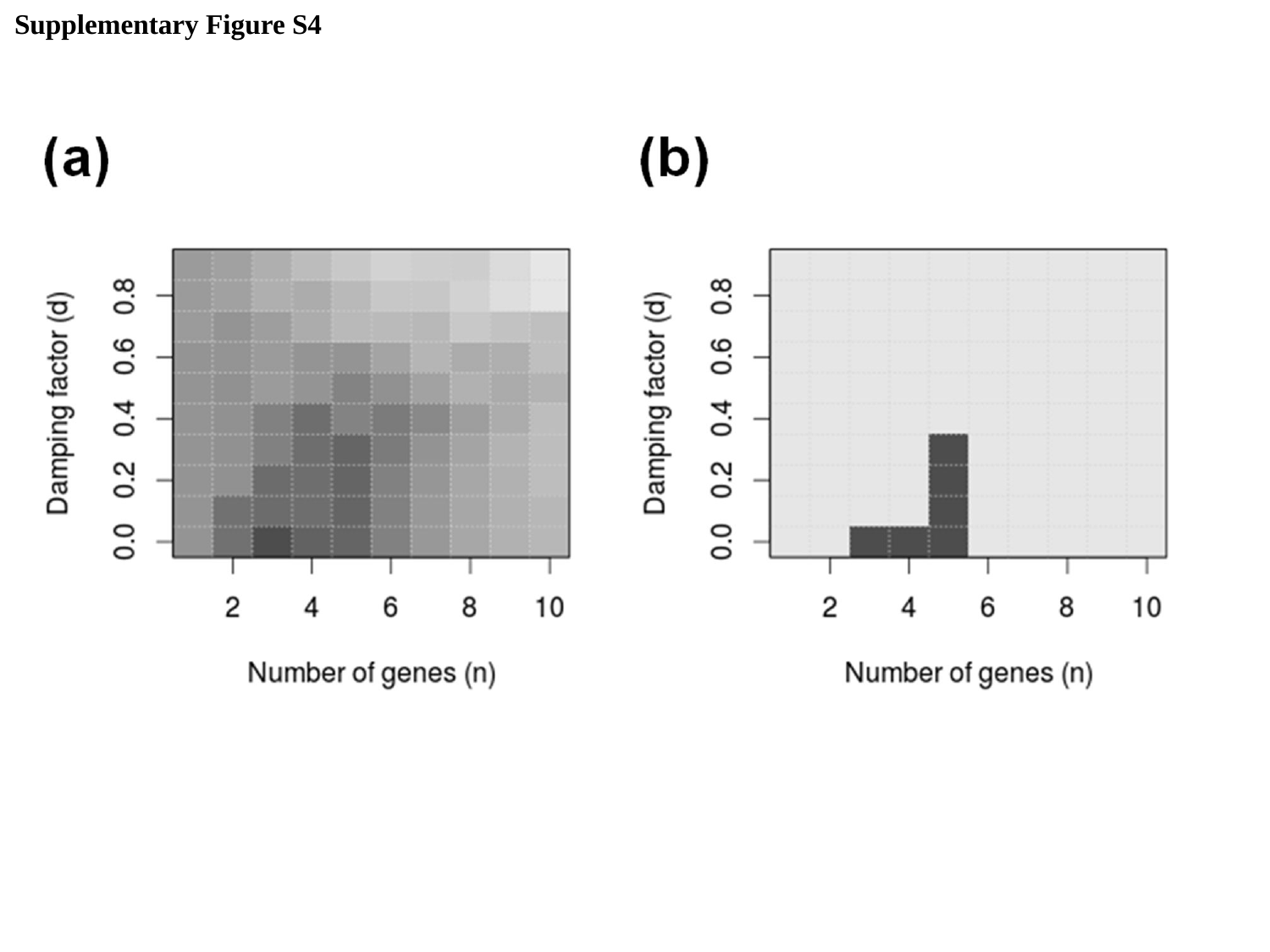

Supplementary Figure S4

## Slide 5
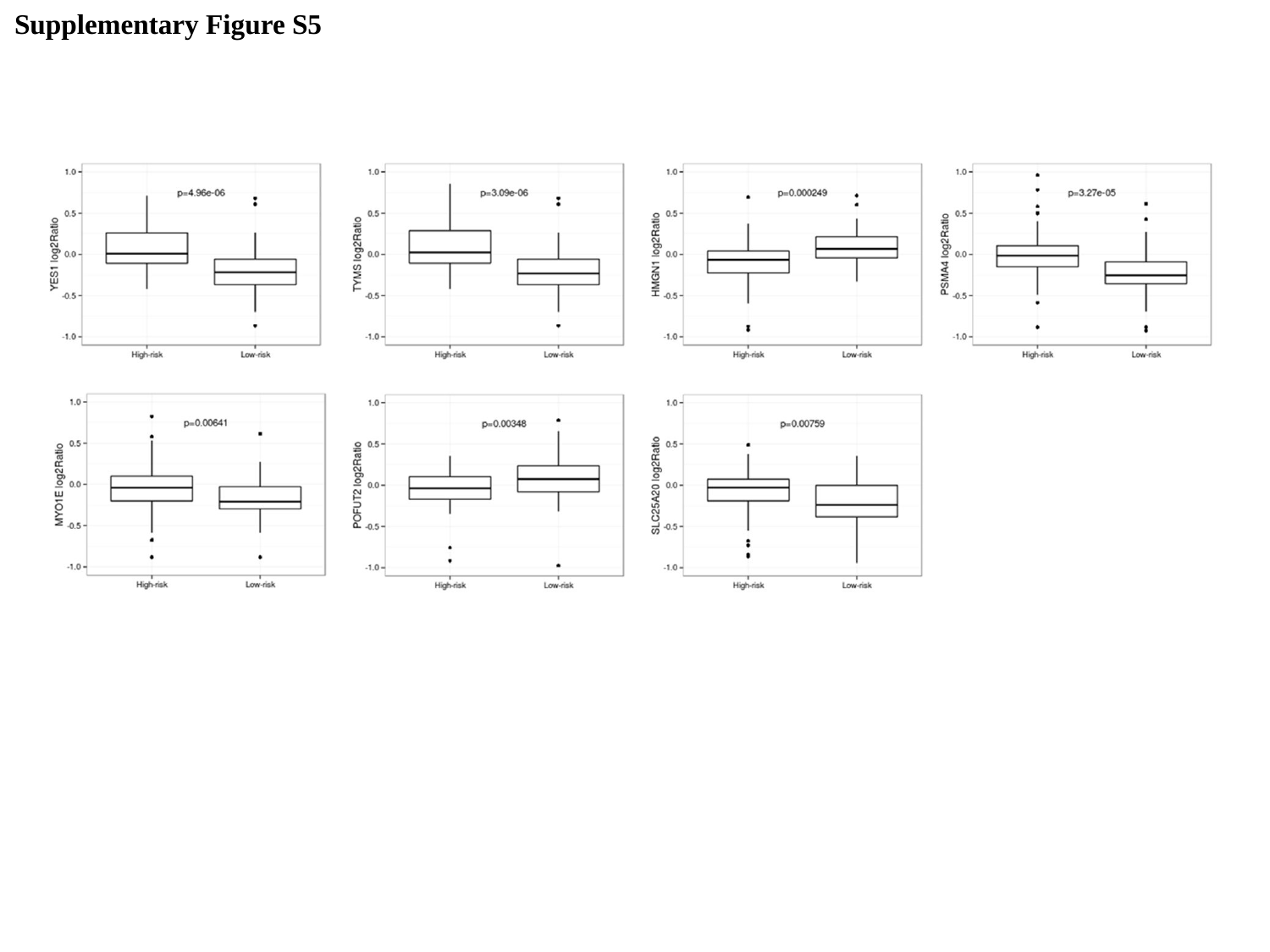

Supplementary Figure S5

## Slide 6
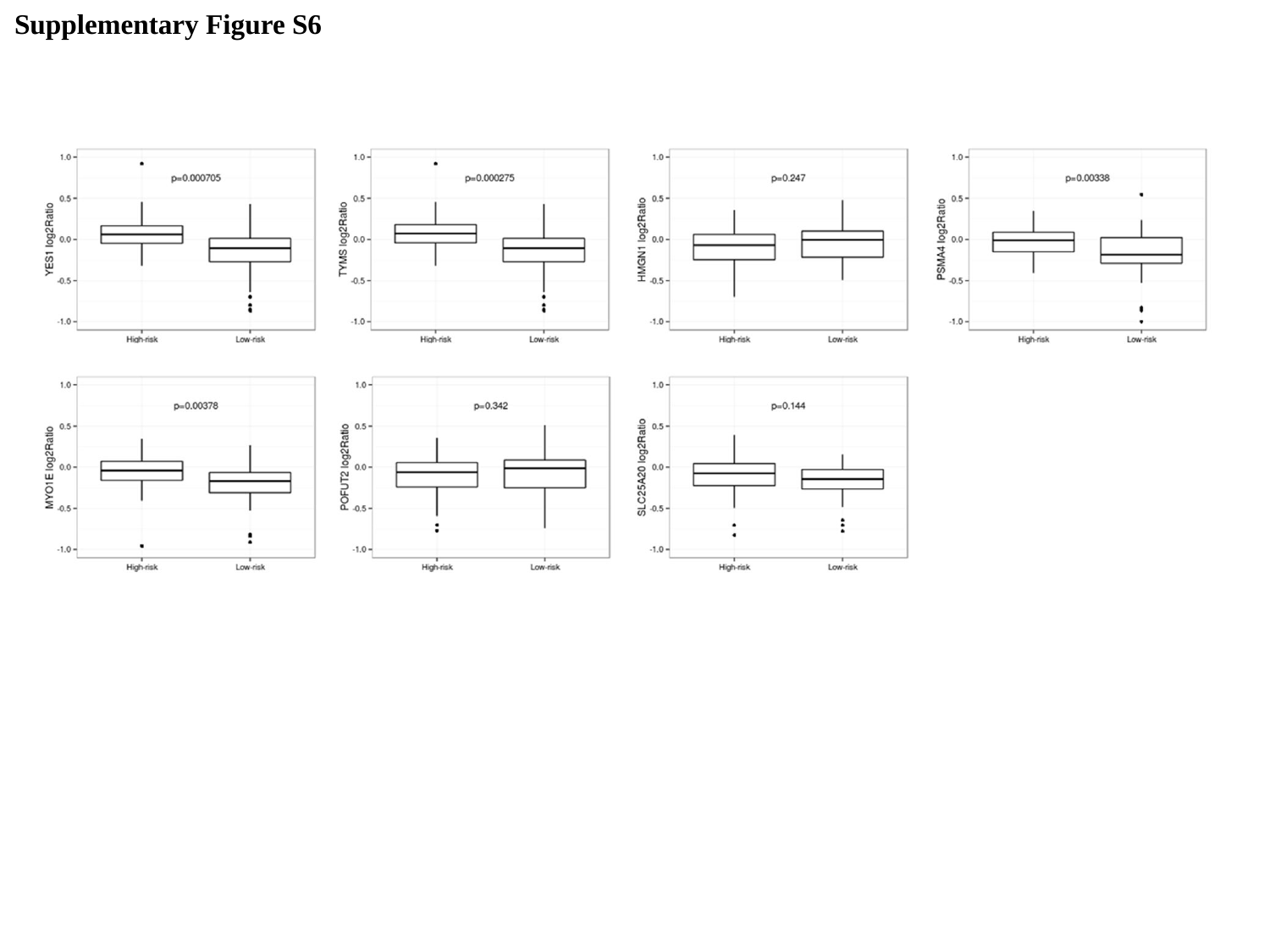

Supplementary Figure S6

## Slide 7
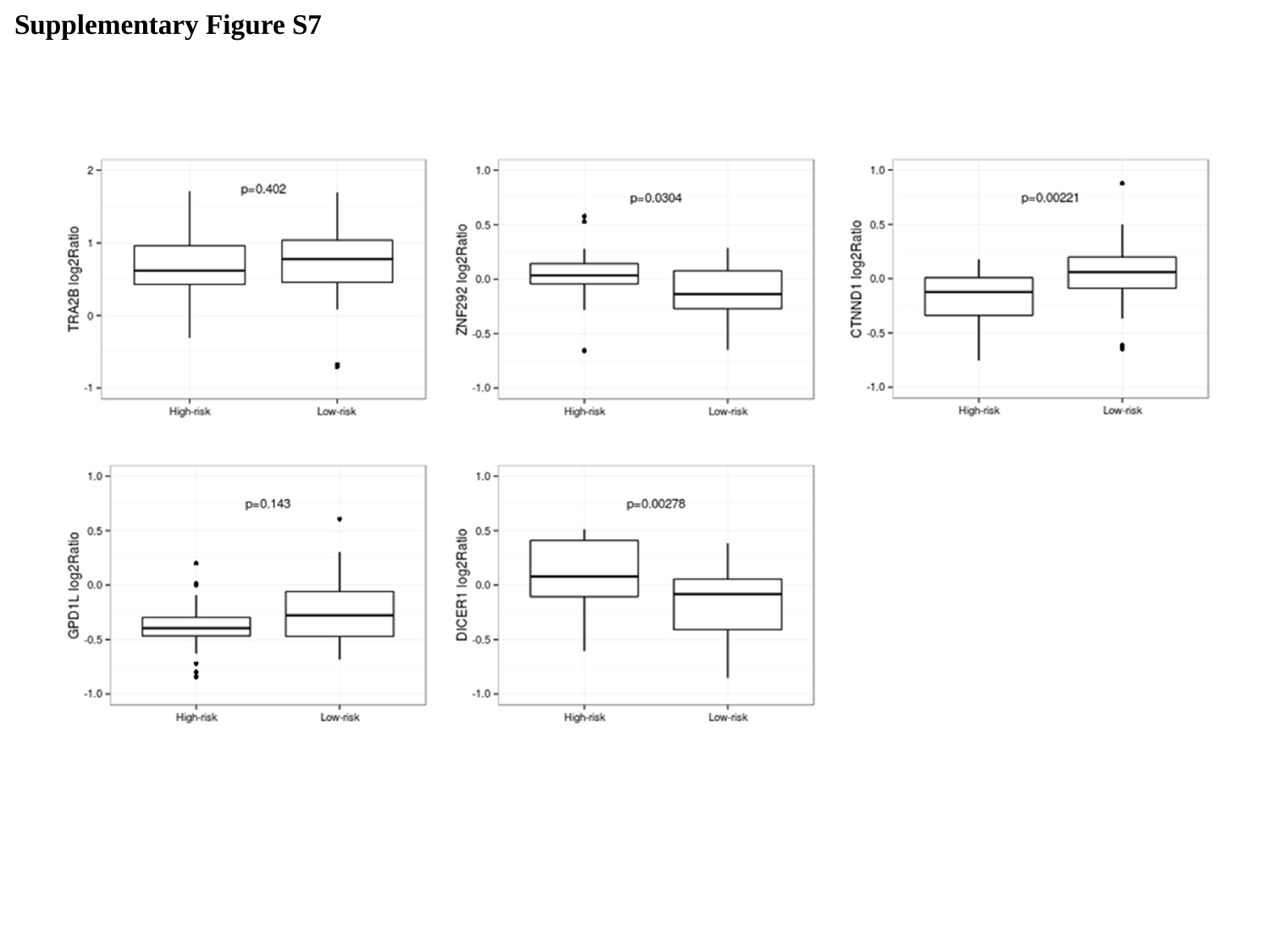

Supplementary Figure S7

## Slide 8
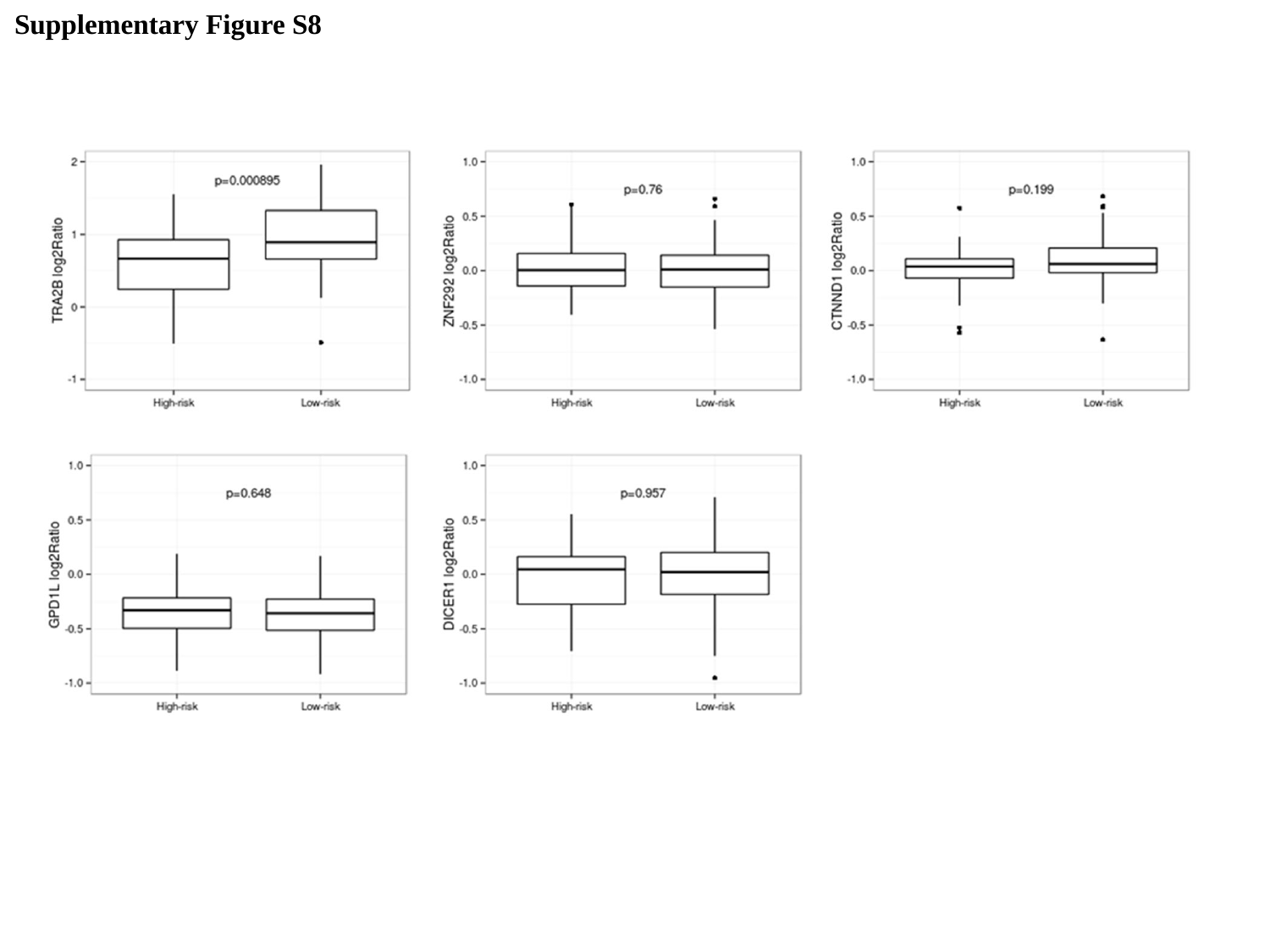

Supplementary Figure S8

## Slide 9
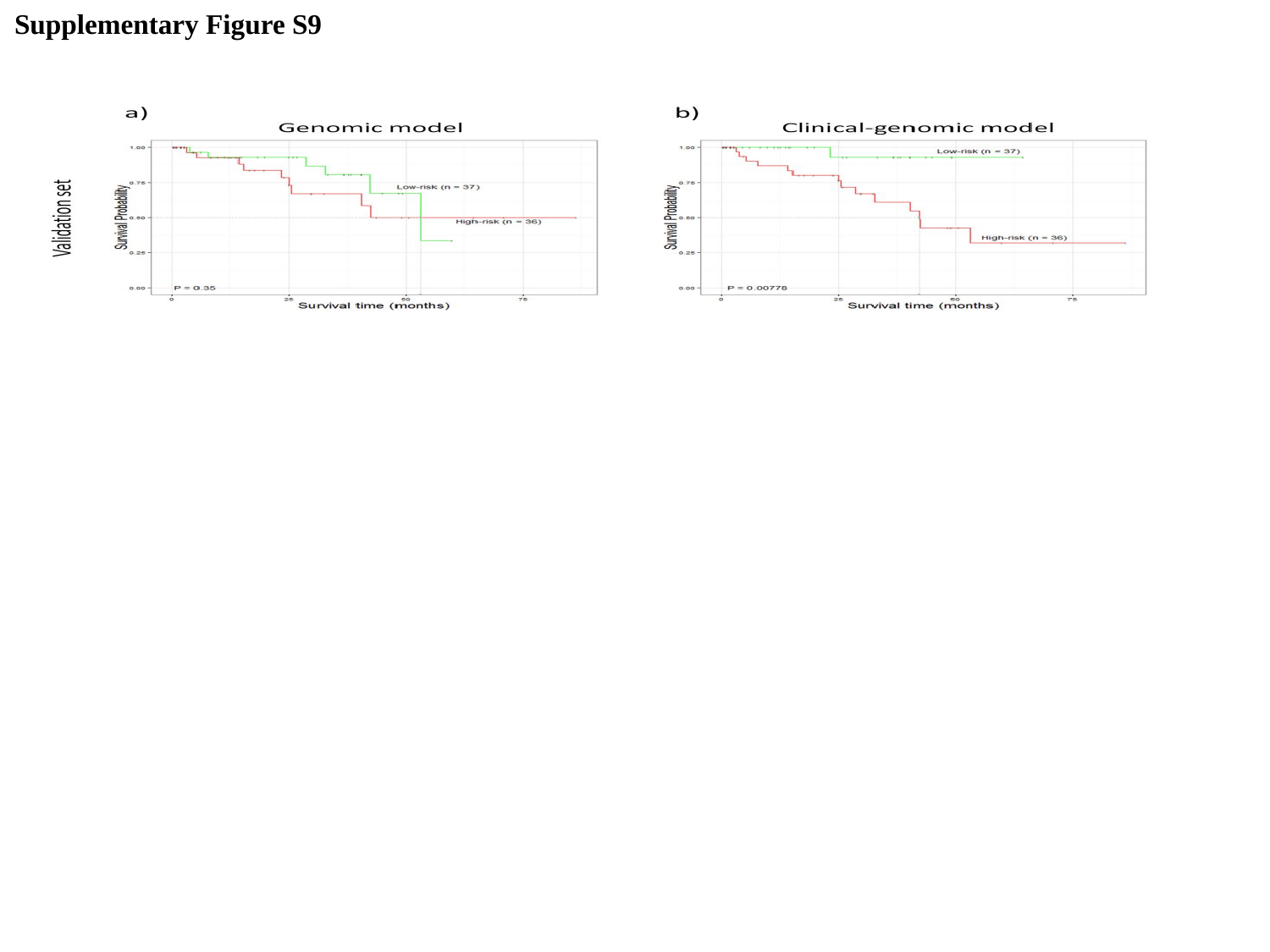

Supplementary Figure S9

## Slide 10
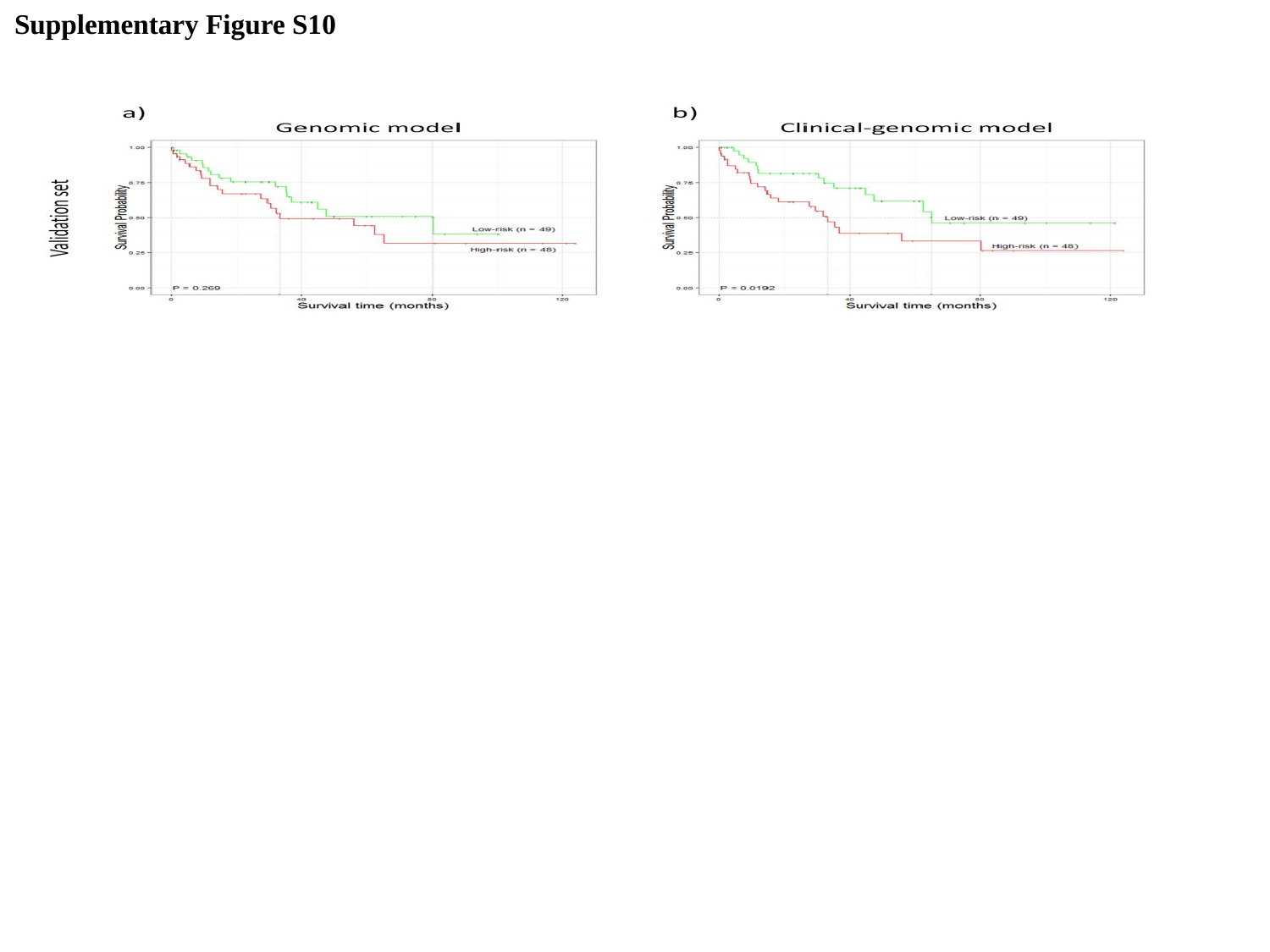

Supplementary Figure S10
